# Application of Continuous Wavelet Transform and Convolutional Neural Network in Decoding Motor Imagery Brain-Computer Interface

**DOI:** 10.3390/e21121199

**Published:** 2019-12-05

**Authors:** Hyeon Kyu Lee, Young-Seok Choi

**Affiliations:** Department of Electronics and Communications Engineering, Kwangwoon University, Seoul 01897, Korea; skgusrb12@kw.ac.kr

**Keywords:** brain-computer interface (BCI), electroencephalography (EEG), motor imagery (MI), continuous wavelet transform (CWT), convolutional neural network (CNN)

## Abstract

The motor imagery-based brain-computer interface (BCI) using electroencephalography (EEG) has been receiving attention from neural engineering researchers and is being applied to various rehabilitation applications. However, the performance degradation caused by motor imagery EEG with very low single-to-noise ratio faces several application issues with the use of a BCI system. In this paper, we propose a novel motor imagery classification scheme based on the continuous wavelet transform and the convolutional neural network. Continuous wavelet transform with three mother wavelets is used to capture a highly informative EEG image by combining time-frequency and electrode location. A convolutional neural network is then designed to both classify motor imagery tasks and reduce computation complexity. The proposed method was validated using two public BCI datasets, BCI competition IV dataset 2b and BCI competition II dataset III. The proposed methods were found to achieve improved classification performance compared with the existing methods, thus showcasing the feasibility of motor imagery BCI.

## 1. Introduction

Brain-Computer Interface (BCI) translates brain signals into an interpretable output without the direct use of peripheral nerves and muscles. The primary purpose of BCI is to create a communication system through brain signals without physical movement for people with severe motor disabilities [1]. Non-invasive BCI model consists of a variety of paradigms on the basis of experimental processes and types of electroencephalography (EEG) recordings. As representative models, event-related potential (e.g., P300), steady-state visual evoked potential (SSVEP), and motor imagery (MI) have attracted attention in the BCI research community. Among them, the MI BCI model has been widely used since it can be easily applied to control external devices [2]. The MI BCI approach is increasingly being applied in various fields, including games [3] and assistive technology [4]. However, despite the growing interest, MI BCI has limitations in real-life applications due to the following issues: First, about 20% of BCI users have difficulties controlling the system compared to others, which we term “BCI illiteracy” [5]. Second, even for the remaining BCI users, particularly those with motor disabilities, MI BCI might not offer the best mental option for BCI control [1,6]. Thus, effective improvement of MI BCI performance remains a challenging issue [7]. 

In recent years, various MI studies using EEG recordings have been conducted [8,9]. The conventional method of MI BCI with EEG signals consists of the extraction of hidden features and subsequent classification based on various machine learning methods. The common spatial pattern (CSP) algorithm is one of the most popular feature extraction methods [10,11]. CSP is commonly used to analyze spatial patterns of multichannel MI EEG signals. However, CSP is considerably dependent on the frequency band of EEG signals. To deal with this issue, variant algorithms, which are extended versions of the CSP, such as filter bank CSP (FBCSP) [12] or filter bank regularized CSP (FBRCSP) [13], are presented. In addition, feature extraction methods that reduce the dimension of EEG signals are becoming popular due to their advanced classification performance. Typical examples include principal component analysis (PCA) [14] and independent component analysis (ICA) [15]. Other widely-known methods, such as Short Time Fourier Transform (STFT) and Wavelet Transform (WT) [16], which possess outstanding time-frequency localization characteristics and multi-resolution properties, have been extensively applied to the feature extraction process. These provide the ability to capture dynamic time-frequency properties of MI EEG signals.

To classify MI EEG signals, a variety of machine learning algorithms have appeared in the literature [17]; they include support vector machine (SVM) [18], linear discriminant analysis (LDA) [19,20], and restricted Boltzmann machines (RBM) [21]. The deep neural network (DNN) approach has recently shown excellent classification performance in this field. The convolutional neural network (CNN) is one of the most famous methods in the DNN model and has various applications. It has successfully achieved object detection tasks [22] and text recognition [23,24]. A recent study by Tabar and Halici [25] presented a classification model based on STFT and 1D CNN, which made use of the 1D CNN with a one-dimensional kernel for images generated by using STFT for MI EEG signals. However, STFT has difficulty in generating images with high quality information about signals due to the trade-off between time and frequency resolution. Thus, this might degrade the classification performance of MI BCI. 

To address this issue, we propose an advanced MI EEG-decoding method using continuous wavelet transform (CWT) and the subsequent 1D CNN with low computational complexity. In order to alleviate the limitation of STFT, a new MI EEG image is formed by CWT with three features (time, frequency, and electrode), which contain a highly informative spectrum without loss of time and frequency features in EEG signals. The input image is composed of the frequency domain with distinct electrodes in the horizontal axis and the time domain in the vertical axis. It is widely known that the characteristics of MI EEG signals are mainly reflected in two frequency bands of EEG signals, i.e., mu (μ)-band (8–13 Hz) and beta (β)-band (13–30 Hz). By utilizing CWT, images using the power spectrum of EEG signals—time, frequency, and electrode information—are obtained. Thus, it is more capable of detecting specific MI patterns in EEG signals compared to Fourier transform. In addition, the use of 1D CNN leads to efficient discrimination of the MI-related patterns that are shown as temporal variations in mu and beta bands. The performance of the proposed method was validated using a public BCI competition dataset [26].

The rest of this paper is organized as follows: Section 2 introduces the experimental datasets used in this study and methodology of the proposed method. The results are presented in Section 3. Section 4 concludes this work. 

## 2. Method

### 2.1. Motor Imagery EEG Datasets

Among the datasets provided by BCI competitions, we used the BCI competition IV dataset 2b [27,28] and the BCI competition II dataset III [29] for validation. Both datasets consist of left and right hand MI, and EEG signals were recorded at C3, Cz, and C4 channels.

As shown in Leeb et al. [27], the first MI EEG dataset, i.e., BCI competition IV dataset 2b, is composed of EEG signals from nine healthy subjects with a sampling frequency of 250 Hz. Each subject consists of two sessions without feedback, three sessions with online smiley feedback, and a total of five sessions. In this study, we utilized previously studied three sessions in whole sessions. In the first two sessions, after 2 s from the beginning on the fixation cross in the 3 s interval, short acoustic stimulus indicates the start of the trial. A visual cue, which is an arrow pointing to the left and right hand, appears for 1.25 s. The subject then performs the MI task on the hand corresponding to the visual cue for 4 s. In the last session, the scheme of the experiment is similar to the preceding sessions. However, the difference is that the visual cue and MI task parts in the preceding sessions were replaced by cue and smiley feedback. The smiley feedback changed to green when user feedback moved in the correct direction; else, it turned to red. The number of trials in each session is 120, 120, and 160, respectively.

The second MI EEG dataset, i.e., BCI competition II dataset III, consists of one subject and 7 runs with 40 trials for each run, which was sampled at 128 Hz. During acquisition of the EEG signals, the subject starts the MI experiment and rests for 2 s. After that, the acoustic stimulus is activated to indicate the beginning of the trials. Then, a cross ‘+’ was displayed for 1 s, and the hand MI task, depending on where the arrow is pointed, is carried out from 3 s to 9 s. The details of the datasets are summarized in Table 1. 

### 2.2. Motor Imagery EEG Image Form Using Continuous Wavelet Transform

We developed a new two-dimensional image by extracting MI features that appear in a specific frequency band during the MI task. The resulting image was obtained through the time-frequency representation of the MI EEG signals. STFT, which is widely used in the time-frequency representation, is ineffective in interpreting MI EEG signals because of a trade-off in resolution between time and frequency. When the size of the window in STFT is short, it results in good time and poor frequency. A wide window offers the opposite results. To resolve this problem, we make use of continuous wavelet transform (CWT) [30] to develop an image of the EEG signal. CWT and Fourier Transform (FT) have similar methods. FT yields correlation coefficients between the original signal and a sinusoidal signal. Similarly, CWT obtains correlation coefficients between the original signal and a mother wavelet. However, unlike the FT, where the signal is decomposed into a frequency domain, CWT assigns the signal to a time-frequency domain by controlling the shape of the mother wavelet. Here, the shape of the mother wavelet is controlled by scaling and shifting parameters. The mathematical formula of CWT is given in Equation (1):

(1) $$\mathrm{CWT}\left(\omega ,s\right)=\frac{1}{\sqrt{\left|s\right|}}\int^{\;}x\left(t\right)\psi \left(\frac{t-\omega }{s}\right)dt$$

where x(t) is MI EEG signal in this paper, ψ is the mother wavelet, ω denotes a time shifting parameter or translation, and s denotes a scaling parameter. CWT(ω,s) represents the correlation coefficients of CWT. The MI EEG signal x(t) can be recovered by an inverse CWT, as following:

(2) $$x\left(t\right)=\frac{1}{C}\int^{\;}\int^{\;}\mathrm{CWT}\left(\omega ,s\right)\frac{\psi_{\omega ,s}\left(t\right)}{{\left|s\right|}^{3/2}}dsd\omega$$

where *C* indicates the normalization constant, which depends on the choice of wavelet.

Unlike STFT with a constant window function, CWT, with a smooth analytical mother wavelet, is capable of identifying the dynamic frequency properties over MI EEG signals at different scales. The CWT coefficients in Equation (1), by applying various scales and translations to the mother wavelet, reflect the similarity of the signal to the wavelet at the current scale. We use three types of mother wavelet, i.e., Morlet, Mexican hat, and Bump wavelets, provided in MATLAB.

The mathematical expressions of Morlet, Mexican hat, and Bump mother wavelets are given by Equations (3)–(5), respectively [31,32,33]. First, as shown in Equations (3) and (4), Morlet wavelet originated from a Gaussian function, while the Mexican hat wavelet, which is also called the Ricker wavelet, is a special case of the second derivative of a Gaussian function. Next, as another type of mother wavelet different from the Gaussian function-based ones, Bump wavelet with scale s and window w is defined in the Fourier domain as Equation (5):

(3) $$\psi_{\mathrm{Morl}}\left(t\right)=e^{2\pi it}e^{-t^{2}/2\sigma^{2}}=\left(\cos2\pi t+\;i\sin2\pi t\right)e^{-t^{2}/2\sigma^{2}}$$

(4) $$\psi_{\mathrm{Mexh}}\left(t\right)=\left(1-\frac{t^{2}}{\sigma^{2}}\right)e^{-t^{2}/2\sigma^{2}}$$

(5) $$\psi_{Bump}\left(sw\right)=e^{\left(1-\frac{1}{1-{\left(sw-\mu \right)}^{2}/\sigma^{2}}\right)}\chi \left[\mu -\sigma ,\mu +\sigma \;\right]$$

where $\psi_{\mathrm{Morl}},\;\psi_{\mathrm{Mexh}}$, and $\psi_{Bump}$ denote Morlet, Mexican hat, and Bump mother wavelets, respectively. The parameter σ plays a role in transshaping the mother wavelet. μ in Equation (5) admits the peak frequency defined by $sw_{\psi }∶=\arg\underset{sw}{\max}\left|\psi_{Bump}\left(sw\right)\right|$ and χ denotes the indicator function.

CWTs with three mother wavelets are employed to a duration of 2 s of the MI EEG signals. We set the frequency range of CWT—from a minimum frequency of 0.1 Hz to a maximum frequency of 50 Hz. Then, we extract the time-frequency image of the mu and beta bands from the overall frequency range. The image of the MI EEG signal is obtained by the following two methods.

First, an input image was obtained from the CWT results of the mu and beta bands. The sizes of the image extracted were 26 × 500 and 37 × 500, respectively. In order to prevent the extracted features from being biased by one dominant frequency band, both bands were resized to have similar size using the cubic spline interpolation method. The deformation of the input image is conducted not only on the frequency axis, but also on the time axis. In the labeled 2 s MI task, the smallest part of the output spectrum of the 0.5 s interval was extracted from the mu-band image. Then, the obtained samples corresponding to 0.5 s were resized to 32 samples by using the cubic spline interpolation method. Subsequently, we obtained a MI EEG image with $N_{f}=31$ and $N_{t}=32$ for one electrode by combining samples generated from the mu and beta bands. The same procedure was repeated for three electrodes, i.e., C3, Cz, and C4, and the resultant three MI EEG are stacked as one MI image. As a result, an MI EEG image has a size of $N_{v}$ × $N_{t}$, where $N_{v}=\;N_{f}\;$× 3. This overall process is carried out for the three mother wavelets.

Second, we make use of a time-frequency image of only the mu-band in the frequency axis. Similar to the previous method, the modification process of the MI EEG image on the time axis is carried out. However, in this method, the frequency axis is not resized to avoid loss of frequency information. The MI EEG image constructed by the proposed method is used as an input to the proposed CNN architecture.

### 2.3. Convolutional Neural Networks Architecture

We conduct MI task recognition based on a variant of CNN. The conventional CNN has shown considerable performance for 2D image classification. CNN consists of input, output, and several hidden layers, which contain several pairs of convolutional-pooling layers and a fully connected layer. The standard CNN extracts the features of the image through the 2D kernel in the convolutional layer and subsampled them to a smaller size in the pooling layer. The reduced image is then classified in the fully connected layer. 

It is to be noted that our input MI EEG image is different from the existing input data format for CNN. Since the input image contains three specific details (time, frequency, and electrode locations), our aim is to classify the hand MI tasks through features in the vertical axis corresponding to the electrode and frequency, whereas the time axis (horizontal axis) is not of critical interest. Therefore, we propose a new MI classification method based on CNN with 1D kernels rather than standard 2D kernels to capture the features of frequency and electrode location on the same time axis.

**Figure 1:**
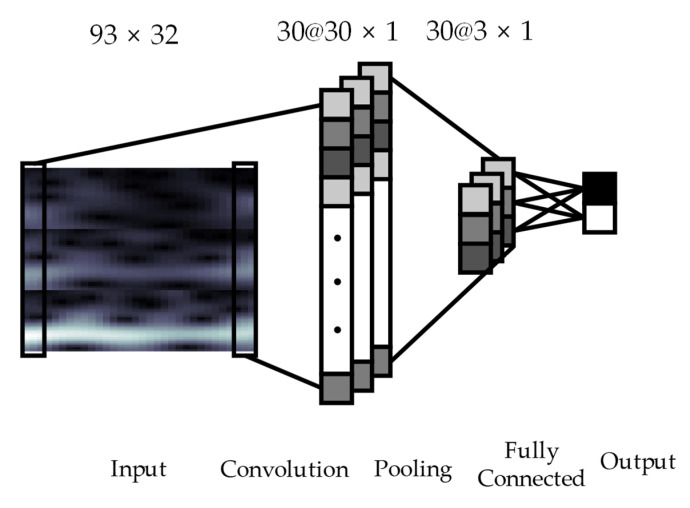
Convolutional Neural Network architecture consists of a convolutional layer with 93 × 3 kernels, a max-pooling layer with sampling factor of 10, and a fully connected layer. The input image with 93 × 32 is passed through the proposed neural network. In the convolution layer, 30 kernels are convolved with the input image. Through a pooling procedure, each feature map is shrunk to 3 × 1. Refer to Section 2.3 for more details.

The proposed CNN architecture with four layers is shown in Figure 1. The input layer is the 2D input MI EEG image with a size of 93 × 32. The input layer is followed by the second and third hidden layers, which consist of one convolutional layer and one max-pooling layer. The following fourth layer is a fully connected layer that differentiates between left or right hand MI tasks. In the convolution layer, the input image is convolved using $N_{F}=30$ kernels with a size of 93 × 3 for the same time axis. The output of convolution between an input image and a kernel is given by Equation (6):

(6) $$y_{i}^{k}=f\left(a\right)=f\left({\left(W^{k}*x\right)}_{i}+b_{k}\right)$$

where x is an input image, $W^{k}$ is a convolution kernel, $b_{k}$ is a bias for k = 1, 2, …, $N_{F}$, and i = 1, 2, …, $N_{t}-2$. As a result, the output applied with stride 1 in the convolutional layer yields 30 feature maps with a size of ($N_{t}-2$) × 1. f(⋅) denotes an activation function; here, the rectified linear unit (ReLU) function is used. The ReLU function is carried out between the convolutional layer and max-pooling layer by the following Equation (7):

(7) $$f\left(a\right)=\mathrm{ReLU}\left(a\right)=\max\left(a,0\right)=\{\begin{array}{l} a,\;\;\mathrm{if}\;a>0\\ 0,\;\;\mathrm{otherwise} \end{array}$$

where a is defined in Equation (6).

The output of the convolutional layer with size of ($N_{t}-2$) × 1 is applied to the input of the max-pooling layer. The max-pooling is carried out with a sampling factor of 10 and zero padding is not used. Therefore, the size of the output of the convolutional layer is subsampled to 3 × 1 dimension for 30 kernels by the max-pooling layer. Finally, the fully connected layer is performed to classify two classes, i.e., left and right hand MI tasks. In this approach, the labeled training data is used for training of the proposed CNN model and the classification error is calculated as the difference between the CNN output and target data. The weights of the neural network during training are updated by the back-propagation algorithm, which is conducted using gradient descent to minimize errors. Using the trained weights of the neural network, MI classification performance with the test MI EEG signals is computed. 

In the proposed method, we make use of the continuous wavelet transform to produce the transient EEG image with improved time and frequency resolution. The use of a one-dimensional convolution neural network results in the improved discrimination capability of temporal variations of motor imagery patterns of an EEG image. In addition, since the neural network consists of not only a one-dimensional kernel to extract MI patterns of the input image but also shallow layers compared to conventional models, it has the advantage of low computation complexity in training. Since the proposed method utilizes an input image with time, frequency, and electrode information, the training of the neural network is robust to variations or abnormal patterns of MI EEG signals.

## 3. Results

### 3.1. Quantification of the Event-Related Desynchronization/Event-Related Synchronization Pattern

In the literature, it has been widely known that MI features are reflected in the mu-band (8–13 Hz) and beta-band (13–30 Hz) of EEG signals [34,35]. In the case of imagination of left and right motor movements, the power decrease of mu and beta bands of EEG signals, named event-related desynchronization (ERD), is observed in the contralateral brain region. In addition, the phenomenon in which the power of both frequency bands of the EEG signals is restored after the MI tasks is called event-related synchronization (ERS). To reflect ERD and ERS, we utilize a method to quantify the ERD/ERS patterns of the MI tasks done in this work [34].

The ERD/ERS patterns are reflected as a variation of power in the MI EEG signal, compared to a reference interval, prior to the start of motor movement imagery. Firstly, each MI EEG channel is averaged over all subjects and trials in the dataset. The ERD/ERS patterns are then calculated as the rate of the change of power with respect to the reference signals, which are given in Equations (8)–(10) [36]:

(8) $${\mathrm{EEG}}_{avg\left(j\right)}=\frac{1}{N}\overset{N}{\underset{i=1}{\sum }}s_{ij}^{2}$$

(9) $${\mathrm{EEG}}_{ref}=\frac{1}{k}\overset{t+k}{\underset{j=t}{\sum }}{\mathrm{EEG}}_{avg\left(j\right)}$$

(10) $$\mathrm{ERD}/\mathrm{ERS}\;\left(\%\right)=\left(\frac{{\mathrm{EEG}}_{avg\left(j\right)}-{\mathrm{EEG}}_{ref}}{{\mathrm{EEG}}_{ref}}\right)\times 100\left(\%\right)$$

where N is the total number of trials and $s_{ij}$ is the *j*th sample of the *i*th trial of the bandpass filtered MI EEG signals. ${\mathrm{EEG}}_{avg\left(j\right)}$ is the average power of MI EEG signals for all trials. ${\mathrm{EEG}}_{ref}$ is the average power of MI EEG signals measured on the reference interval.

**Figure 2:**
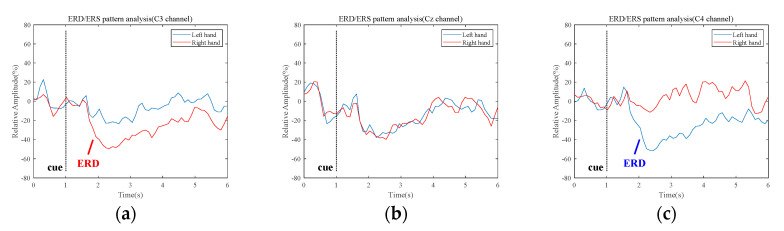
Event-related desynchronization/Event-related synchronization patterns over each channel (C3, Cz, and C4) during the hand Motor Imagery tasks in mu-band: (**a**) C3 electrode; (**b**) Cz electrode; (**c**) C4 electrode.

To extract a reliable MI task interval, we detect the ERD/ERS patterns in typical motor movement related frequency bands, e.g., mu-band (8–13 Hz), beta-band (13–30 Hz), and combined mu and beta band (8–30 Hz). In general, ERD/ERS patterns are distinctly observed in the motor cortex region. Figure 2 shows the ERD/ERS patterns in mu-band, which are recorded from C3, Cz, and C4 electrodes, respectively. The relative amplitude indicates the values calculated by Equation (10) across all subjects in the first dataset. Details on the dataset are described in Section 2.1. As shown in Figure 2, the ERD patterns occur bilaterally during the MI tasks (second 2–5), which are lateralized to the contralateral hemisphere. However, ERS patterns on the motor cortex are not observed clearly in mu-band. The ERD and ERS patterns in the Cz channel are not clearly distinguished, compared to other electrodes. Therefore, in order to extract a common MI task interval from datasets used in this paper, we chose a 2 s MI task interval (0.5 s~2.5 s after the visual cue is displayed), where the ERD patterns actively appear.

### 3.2. Classification Results

**Figure 3:**
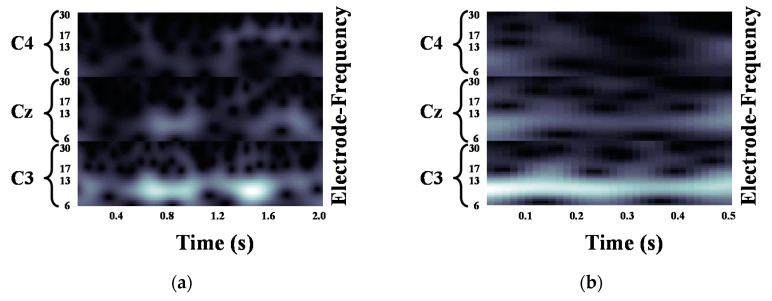
Motor Imagery EEG image for left hand Motor Imagery task using Morlet wavelet: (**a**) size of 93 × 500; (**b**) size of 93 × 32.

**Figure 4:**
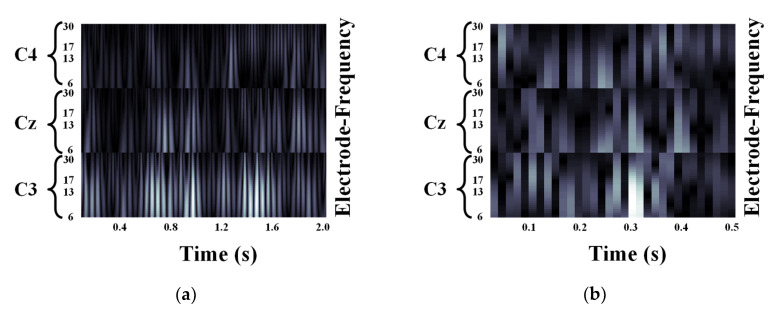
Motor Imagery EEG image for left hand Motor Imagery task using Mexican hat wavelet: (**a**) size of 93 × 500; (**b**) size of 93 × 32.

**Figure 5:**
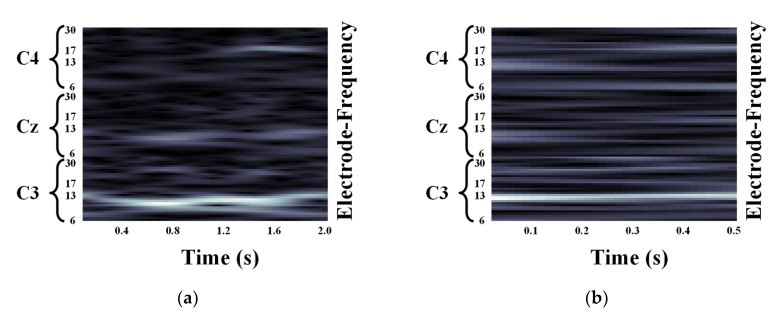
Motor Imagery EEG image for left hand Motor Imagery task using Bump wavelet: (**a**) size of 93 × 500; (**b**) size of 93 × 32.

**Figure 6:**
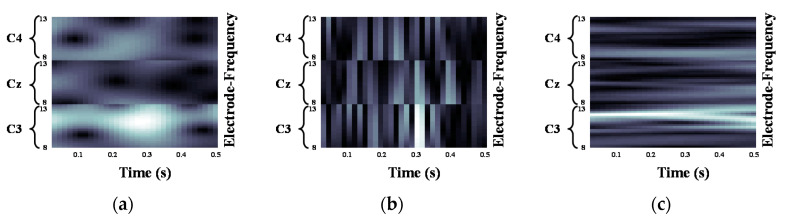
Motor Imagery EEG image with mu-band for left hand Motor Imagery task: (**a**) Morlet wavelet; (**b**) Mexican hat wavelet; (**c**) Bump wavelet.

We validated the classification performance of the proposed CNN for two input image types and three mother wavelets. Here, we show MI EEG images for left hand MI tasks by utilizing CWTs to extract mu and beta bands or only mu-band of EEG signals. Figure 3, Figure 4 and Figure 5 show the MI EEG images generated by the first method, i.e., using the mu and beta bands, for a left hand MI task using three distinct mother wavelets, i.e., Morlet, Mexican, and Bump wavelets, respectively. In each figure, the left figure denoted by (a) shows the resized image on the frequency axis, and the right figure denoted by (b) denotes the resized image both on the frequency and the time axes. Compared to FT and STFT, the use of CWT helps reveal ERD patterns of the mu-band from an EEG input image more clearly without loss of information in terms of time and electrode-frequency due to its superior time-frequency resolution. Figure 6a–c show the MI EEG images generated by using only the mu-band and three mother wavelets, respectively. As stated before, ERD patterns are shown in the MI EEG recorded from C4 electrode contralaterally in case of left hand MI tasks. Thus, in the figures, the ERD patterns are represented as a decrease of mu-band power in the C4 electrode, compared to other electrodes, i.e., Cz and C3. As a result, as shown in Figure 3, Figure 4, Figure 5 and Figure 6, the generated EEG images depict the ERD patterns of mu-band EEG signals; the mu-band power of the C4 electrode is lower than those of other electrodes, regardless of mother wavelets.

Table 2 and Table 3 indicate the average accuracy and standard deviation across all subjects in the two datasets used in this paper. We compared classification performance with the previous CNN-based MI classification method, which used STFT and CNN [25]. The first dataset, BCI competition IV dataset 2b, is comprised solely of the labeled training set. To evaluate the accuracy of the dataset, we divided it into training and test sets for each subject using 10-fold cross-validation. Thus, 90% of the total 400 trials per subject was randomly selected as the training set and the rest as the test set. This process was repeated 10 times and a total of 100 test sets were produced to reduce the effect of within-subject variation.

The test sets were evaluated by the proposed CNN, which is described in Section 2.3. The CNN structure consists of one convolutional layer, one max-pooling layer, and one fully connected layer. CNN was trained by using a batch training method with a batch size of 50 for 300 epochs. Data normalization was applied to the input MI EEG image in an interval between 0 and 1.

Table 2 shows the classification performance depending on input image types using the BCI competition Ⅳ dataset 2b. As can be seen, for the first input MI EEG image type with the mu and beta band, the proposed method yields average accuracy of 83.0%, 81.2%, and 81.7% for Morlet, Mexican hat, and Bump mother wavelet, respectively. Since the previous study using STFT has 74.8% accuracy, the proposed method achieves an improvement for classification of MI tasks. In addition, the proposed method with an input image using mu-band outperformed the STFT-based method, while it is comparable with the use of an input image using the mu and beta bands. For distinct mother wavelets, using the Molet wavelet for combined mu and beta bands results in the best classification accuracy.

Table 3 indicates the classification performance using the second dataset, i.e., BCI competition II datasets III. This dataset consists of a labeled training set and an unlabeled test set. The number of trials for each set is 140. This dataset with sampling rate of 128 Hz was likewise selected for the samples for 2 s after 0.5 s from the start of the cue appearance, as described in Section 2.2. The parameters used in the CNN model are the same as those applied to the first dataset. The results in Table 3 show that the proposed method is superior to the STFT-based method for both input image types. Note that STFT for an input image using mu-band is not available to compute accuracy, whereas the proposed method achieves comparable performance with the use of both mu and beta bands.

## 4. Conclusions

In this study, we propose a new continuous wavelet transform and convolutional neural network based decoding scheme to classify motor imagery tasks. The proposed method is comprised of two stages: image generation using continuous wavelet transform for motor imagery EEG signals and motor imagery tasks classification using the proposed one-dimensional convolutional neural network. By employing continuous wavelet transform, highly informative input motor imagery EEG image with time, frequency, and electrode location is generated. We confirm that the resultant motor imagery EEG image contains event-related desynchronization patterns, along with frequency and electrode location. Next, using the motor imagery EEG image as input, a one-dimensional convolutional neural network with four layers is developed to decode the two distinct motor imagery tasks. The network aims to capture the one-dimensional dynamics of event-related desynchronization patterns of the input motor imagery EEG image.

In the proposed method, the use of continuous wavelet transform yields a more detailed representation of motor imagery-related EEG patterns, compared to Fourier transform-based methods. In addition, by utilizing a one-dimensional convolutional neural network, it is capable of discriminating between temporal variations of EEG signals of motor imagery tasks, in terms of specific frequency and electrode. The combinational use of wavelet transform and neural network may lead to the development of advanced signal processing and machine learning tools to analyze EEG signals in motor imagery BCI research.

## Figures and Tables

**Table 1:** Details of the datasets.

Dataset	Subjects	Channels	Trials	Sampling Frequency (Hz)	MI Class
BCI competition Ⅳ dataset 2b	9	C3, Cz, C4	400	250	2 (left/right hands)
BCI competition Ⅱ dataset Ⅲ	1	C3, Cz, C4	280	128	

**Table 2:** Average classification accuracy and standard deviation (%) for BCI competition IV dataset 2b.

Subjects	Accuracy (%) and Standard Deviation						
	STFT [25]	CWT					
		Morlet		Mexican Hat		Bump	
	mu + beta	mu + beta	mu	mu + beta	mu	mu + beta	mu
1	74.5 ± 4.6	85.6 ± 1.3	84.7 ± 1.6	81.8 ± 1.3	81.7 ± 1.6	83.2 ± 1.4	82.4 ± 1.1
2	64.3 ± 2.0	72.8 ± 1.4	72.7 ± 2.0	70.6 ± 2.1	71.9 ± 2.0	73.8 ± 2.1	72.5 ± 2.0
3	71.8 ± 1.6	78.0 ± 1.9	79.5 ± 2.1	76.4 ± 1.8	74.7 ± 2.1	71.5 ± 2.1	73.6 ± 1.8
4	94.5 ± 0.2	95.4 ± 1.0	96.4 ± 0.5	96.0 ± 0.4	95.0 ± 0.9	96.2 ± 0.8	97.4 ± 0.5
5	79.5 ± 2.5	82.6 ± 1.7	79.6 ± 2.1	78.7 ± 1.9	75.6 ± 2.0	81.0 ± 1.0	73.1 ± 1.7
6	75.0 ± 2.4	79.8 ± 2.1	77.9 ± 1.6	75.5 ± 2.2	76.9 ± 1.5	80.6 ± 1.8	81.0 ± 1.3
7	70.5 ± 2.3	82.9 ± 1.2	81.0 ± 1.6	82.1 ± 1.2	81.4 ± 1.8	78.9 ± 2.0	81.7 ± 1.9
8	71.8 ± 4.1	85.0 ± 1.9	85.7 ± 1.7	84.7 ± 1.4	83.5 ± 1.4	83.5 ± 1.5	83.1 ± 1.6
9	71.0 ± 1.1	85.3 ± 1.9	84.9 ± 1.4	84.6 ± 1.2	85.1 ± 1.7	86.6 ± 1.4	84.0 ± 2.2
Mean	74.8 ± 2.3	83.0 ± 1.6	82.5 ± 1.6	81.2 ± 1.5	80.6 ± 1.7	81.7 ± 1.6	81.0 ± 1.6

**Table 3:** Average classification accuracy (%) for BCI competition Ⅱ dataset III. N/A denotes ‘not available’.

Frequency Band	Accuracy (%)			
	STFT [25]	Morlet	Mexican Hat	Bump
Mu + beta	89.3	89.3	90.0	92.9
mu	N/A	91.4	89.2	91.4
